# Fully Covered Self-Expandable Metal Stents for Treatment of Malignant Biliary Strictures due to Pancreatic Carcinoma

**DOI:** 10.4021/gr466w

**Published:** 2012-09-20

**Authors:** Ahmed Abdel Samie, Michael Stumpf, Lorenz Theilmann

**Affiliations:** a Department of Gastroenterology, Pforzheim Hospital, Germany; b Department of Surgery, Pforzheim Hospital, Germany

**Keywords:** Completely covered self-expandable metal stents, Pancreatic carcinoma, Biliary stenosis

## Abstract

**Background:**

Transpapillary stents are used to treat malignant biliary strictures. However, there are different stent types and data are controversial in respect to success and complications. Recently, completely covered self-expandable metal stents (CSEMS) have become available. The aim of this study is to present a consecutive series of CSEMS placed to decompress the bile duct in malignant stenosis due to pancreatic carcinoma and to evaluate the effectiveness, complication rate and extractability of these devices.

**Methods:**

We retrospectively reviewed the courses of 27 consecutive patients who received CSEMS due to malignant biliary strictures because of pancreatic carcinoma regardless of presumed resectability between January 2010 and May 2012 in our endoscopic unit.

**Results:**

A total of 27 patients (12 male and 15 female) were included in the study. The mean age of the patients was 75 years. Endoscopic retrograde cholangiopancreatography (ERCP), endoscopic sphincterotomy (ES) and stent placement were successful at first attempt in all cases. The mean length of the stenosis was 20 mm. In 24 patients (89%) a stent length of 4 cm was sufficient to bridge the stenosis. In three cases a stent length of 6 cm was necessary. Drainage was achieved as monitored by a significant decrease or normalization of bilirubin in all cases (mean bilirubin 8.5 mg/dL and 1.5 mg/dL before and after stent placement respectively), 15 patients underwent surgery with pylorus preserving duodenopancreatectomy. In all patients who underwent surgery stents could be removed during the operation without difficulties. Leakage of the biliodigestive anastomosis occurred in one patient (6.6%). Four (15%) of the 27 patients developed complications related to the endoscopic procedure and/or stent placement respectively (cholecystitis in two patients, stent occlusion in one patient, and post-sphincterotomy bleeding in one patient).

**Conclusion:**

The prolonged patency, extractability, and low complication rate of CSEMS make them an attractive treatment option in patients with malignant biliary strictures due to pancreatic carcinoma regardless of resectability.

## Introduction

A multicenter trial comparing preoperative biliary drainage to early surgery for cancer of the pancreatic head showed that routine preoperative biliary drainage increased the rate of complications significantly [1]. The authors concluded that it should not be used routinely.

Although drainage was primarily successful in 94%, there was a significant rate of cholangitis and a rate of 30% for the need of stent exchange leading to more readmissions. In addition, surgeons often report that they feel that anastomosis of the remaining biliary tract to the jejunal loop is more difficult and hampered because of local inflammation caused by the stent. However, in the trial above, plastic stents have been used. These stents have a narrow diameter which results in a high rate of stent occlusion over time with consequent cholangitis. This problem can partially be overcome using wide-bore stents such as self-expanding metal stents [2]. The disadvantage of these devices, however, is that once positioned they cannot be removed easily without major damage to the biliary duct. Recently, completely covered self-expanding metal stents have become available making removal either endoscopically or during surgery easily possible. In addition, CSEMS seem to have a greater patency rate compared to uncovered self-expandable metal stents, as the covering of the metal stent mesh prevents tumor ingrowth [3].

However data regarding effectiveness and complication rates of CSEMS are contradictory. Siddiqui et al [4] reported a high patency and a low complication rate of these devices and recommends usage of CSEMS as the initial intervention in patients with malignant biliary stenosis, even if surgical resectability is uncertain. Nevertheless, another lately published study documented a low long term patency and a high complication rate of CSEMS [5].

Therefore, we sought to present a consecutive series of CSEMS placed to decompress the bile duct in malignant stenosis due to pancreatic carcinoma and to evaluate effectiveness, complication rate and extractability of these devices.

## Patients and Methods

We retrospectively analyzed the medical records of all patients, who underwent endoscopic retrograde cholangiopancreatography with endoscopic sphincterotomy and placement of completely covered self-expandable metal stents due to biliary obstruction because of pancreatic ductal adenocarcinoma between January 2010 and May 2012 in our unit (Department of Gastroenterology, Pforzheim Hospital, Germany).

Pforzheim Hospital is a 500-bed, academic teaching, tertiary care hospital.

Demographic characteristics of the patients, their clinical, endoscopic, and laboratory findings as well as their cross-sectional imaging were reviewed. All patients who did not undergo surgery (palliative group) were followed up until death. The indication of stent placement was severe cholestasis and/or cholangitis in all cases. ERCP was performed under propofol sedation using a standard duodenoscope. ES was carried out with standard sphinctertome-based techniques using a guide wire and Endo Cut mode (ERBE, Germany). After exact fluoroscopic localization of the stricture and ES, a completely covered self-expandable metal stents (WallFlex RX, Boston Scientific) was placed over a standard guide wire across the stenosis and deployed under endoscopic and fluoroscopic control. Depending on the type of stenosis, a length of either 40 mm with 10 mm diameter or a length of 60 mm with 8 mm diameter was chosen. Antibiotics were administrated only if cholangitis was suspected. All procedures were conducted by four experienced interventional gastroenterologists within two days after hospital admission. At least, one cross-sectional imaging modality (abdominal sonography/US or computed tomography/CT) was carried out prior to ERCP. Staging was completed during the further course with US and CT performed in all cases. Endoscopic ultrasound (EUS) was performed if other imaging findings were inconclusive. The results of EUS were not adversely affected by the previously implanted stents. In the palliative group, the diagnosis of pancreatic carcinoma was confirmed with either CT-guided or US-guided fine-needle biopsy.

### Statistical analysis

End point was death in the palliative group. Patients who underwent surgery were followed up for 30 days postoperatively. All data were collected and analysed on Microsoft Excel for Windows.

## Results

Between January 2010 and May 2012 27 patients (12 male and 15 female) were included in our study. The mean age of the patients was 75 years (Table 1).

**Table 1:** Patient Characteristics

No. Patients	27
Sex (male/female)	12/15
Mean Age (y)	75 (53 - 91)

ERCP, ES and stent placement were successful at first attempt in all cases. In one patient intubation of the bile duct was not successful with standard techniques, and a precut sphincterotomy was preformed enabling selective cannulization of the biliary passages in the same setting. The mean length of the stenosis was 20 mm. In 24 patients (89%) a stent length of 4 cm was sufficient to bridge the stenosis. In three cases a stent length of 6 cm was necessary. Drainage was achieved as monitored by a significant decrease or normalization of bilirubin in all cases (mean bilirubin 8.5 mg/dL and 1.5 mg/dL before and after stent placement respectively).

15 patients underwent surgery with pylorus preserving duodenopancreatectomy within a mean duration of eight days following biliary drainage. Leakage of the biliodigestive anastomosis occurred in one patient (6.6%). This patient died due to a cause not related to the procedure (liver failure). Otherwise, no increased rate of complications or difficulties in creating an anastomosis with the remaining bile duct was reported by our surgeons. The median postoperative duration of hospitalization was 20 days. In 12 patients (44.4%) curative surgery was not possible because of advanced local disease or presence of distant metastasis at presentation. This group (palliative group) was monitored on follow-up until death. Two patients were lost to follow-up. The mean survival time of this group was 3.5 months.

### Complications

In our series four (15%) of 27 patients developed complications related to the endoscopic procedure and/or stent placement respectively (Table 2).

**Table 2:** Complications

Complication	No. Patients
Cholecystitis	2 (7%)
Post-sphincterotomy bleeding	1 (4%)
Stent occlusion (tumor overgrowth)	1 (4% )

Two patients developed cholecystitis (7%) within three days after stent placement.

In both patients the stents had to be removed as it blocked the cystic duct, and antibiotic therapy was administrated leading to rapid resolution of symptoms. In one patient (4%) stent occlusion proximal to the upper end occurred due to tumor overgrowth. The stenosis was successfully bridged with a second metal stent. In one patient clinically significant post-sphincterotomy bleeding occurred 48 hours after ES. Hemostasis was achieved endoscopically via injection therapy with diluted epinephrine.

Post-ERCP pancreatitis leading to prolongation of the hospital stay or requiring the administration of analgesics could not be observed in any patient.

In our series neither stent occlusion due to debris nor stent dislocation occurred.

### Removal of stents

**Figure 1:**
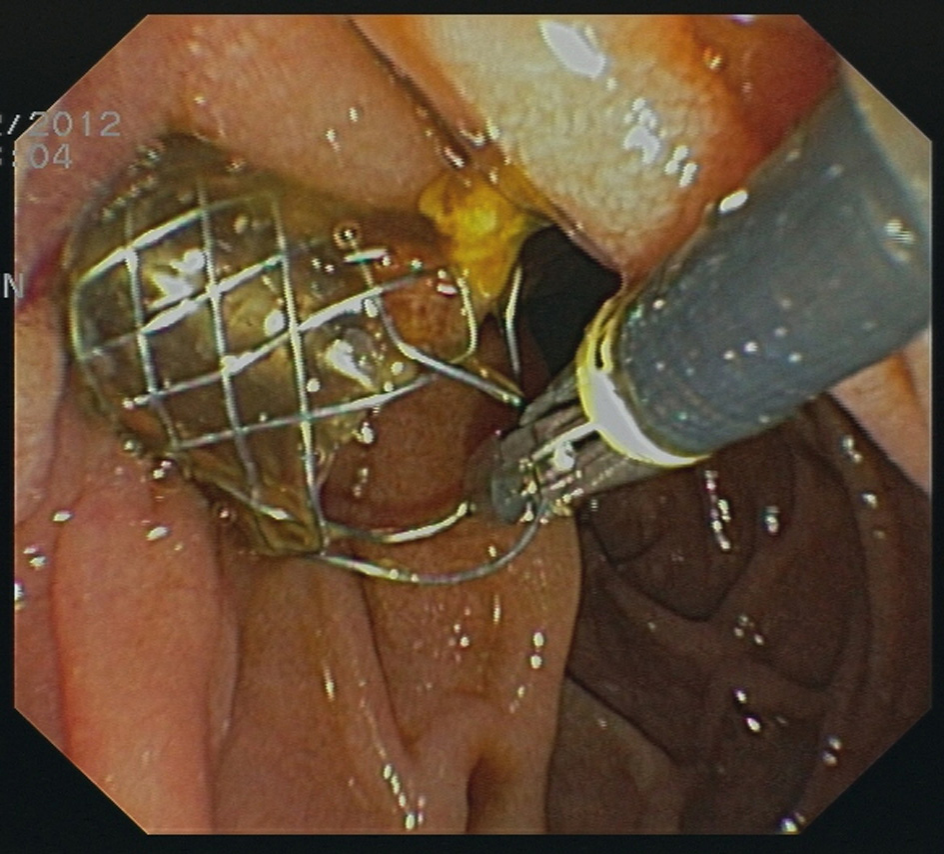
Removal of a CSEMS using a standard forceps.

Two stents were removed endoscopically prior to surgery due to cholecystitis. Removal of stents was easily possible in both cases using standard forceps (Fig. 1).

In all patients who underwent surgery stents could be removed during the operation without difficulties.

## Discussion

Pancreatic cancer is the most common cause of malignant biliary obstruction. Jaundice occurs in 70-90% of the patients during the course of the disease. In the past, plastic stents were used in the first instance in the palliative care to relieve biliary obstruction.

However, preoperative biliary drainage using plastic stents in patients with pancreatic carcinoma has been shown to be inferior to early surgery, and is associated with high rates of serious complications [1].

Compared with uncovered metal stents, covered metal stents have a longer patency and a lower rate of tumor ingrowth [6]. A randomized controlled trial comparing covered and uncovered metal stents in patients with malignant biliary obstruction showed a longer stent patency of covered (304 days) compared with uncovered stents (161 days) [7].

In a further study the mean patency duration of CSEMS was 5.5 months. Moreover, this treatment has the advantage of less costly interventions compared with other treatment options [8]. This is in accordance with another recent report, in which the authors recommend fully covered self-expanding stents as the initial intervention for biliary obstruction even if the surgical respectability status is uncertain [4].

In our retrospective analysis CSEMS was effective to reveal biliary obstruction in all cases.

The internal diameter of 8 to 10 mm of these stents ascertains sufficient bile flow, if bile is very viscous because of previous biliary obstruction. We chose the length of the stent as short as possible to avoid any alteration of the proximal common bile duct. No problems in creating the anastomosis were reported by our surgeons and leakage of the biliary anastomosis was observed in only one case. In patients in whom resection was not intended or could not be performed because of widespread or metastatic disease, stents remained in place on follow-up.

So far, only plastic stents could be removed safely endoscopically if necessary. The disadvantage of these stents, however, is the small internal diameter which predisposes to occlusion by biliary debris. On the other hand, self-expanding metal stents resulted in efficient drainage of biliary obstruction with good bile flow due to the large diameter in contrast to plastic stents. The big drawback of self-expanding metal stents, nevertheless, was that they could not be extracted easily, if at all. With the availability of fully covered self-expandable metal stents this short-coming has been overcome.

In a multicenter study, including 37 patients removal attempts of the CSEMS were successful in all cases [9]. The endoscopic feasibility and safety of stent removal were also documented by other authors [10]. In our series removal of the stents was feasible and safe in all cases, in whom stent explanation was desired (two patients preoperatively due to cholecystitis and 13 patients during surgery). The overall complication rate related to the endoscopic procedure and/or CSEMS placement was 15% in our unit. We observed cholecystitis in two patients (7%). In both patients, the outlet of the cystic duct into the common bile duct was blocked by the stent.

In one study, this complication occurred in 20% of cases if the CSEMS covered the cystic duct [11]. Careful fluoroscopic documentation of the cystic duct origin during ERC with stent placement distal to it may reduce this complication. In strictures at the level of the cystic duct, in which stent placement across the duct is inevitable, a gallbladder stent seems to be an effective strategy to reduce risk of developing cholecystitis after CSEMS placement [5, 11]. This complication can also be controlled by removal of the stent and antibiotic therapy as shown in our two cases. Insertion of a percutaneous cholecystostomy drain represents another effective non surgical treatment option for these patients [5].

Although CSEMS was successfully used to control post endoscopic sphincterotomy bleeding not controlled by other measures as shown by different authors, including the authors of this paper, we observed a clinically significant late bleeding following endoscopic sphincterotomy in one case. Acute cholangitis is an established risk factor for post sphincterotomy bleeding and might have predisposed to bleeding in this patient [12, 13, 14].

Stent migration represents a further complication of CSEMS, especially when placed for treatment of benign biliary strictures. In this setting stent migration seems to be a relevant problem occurring in between 30 and 50% of the cases, although presenting with no clinical consequences in most patients [15, 16]. In malignant biliary strictures however, this complication is rarely seen. In our series stent migration was not observed in any patient.

Despite the retrospective design of our single center study, we conclude that CSMES are an effective treatment option with low complication rates for patients with malignant biliary stenosis due to pancreatic carcinoma. Our results emphasize the need of a large prospective multicenter study comparing different stent types for this indication.

### Conclusion

The prolonged patency, extractability, and low complication rate of CSEMS make them an attractive treatment option in patients with malignant bile duct stenosis due to pancreatic carcinoma regardless of resectability.
